# Drug-related deaths in Scotland 1979–2013: evidence of a vulnerable cohort of young men living in deprived areas

**DOI:** 10.1186/s12889-018-5267-2

**Published:** 2018-03-27

**Authors:** Jane Parkinson, Jon Minton, James Lewsey, Janet Bouttell, Gerry McCartney

**Affiliations:** 1 Public Health Observatory, NHS Health Scotland, Meridian Court, 5 Cadogan Street, Glasgow, G2 6QE UK; 2 Urban Studies, School of Social and Political Sciences, University of Glasgow, 25 Bute Gardens, Glasgow, G12 8RT UK; 3 Health Economics and Health Technology Assessment, Institute of Health and Wellbeing, University of Glasgow, 1 Lilybank Gardens, Glasgow, G12 8RZ UK

**Keywords:** Drug-related deaths, Excess mortality, Scottish effect, Age-period-cohort effects, Scotland, Shaded contour plots, Intrinsic estimator

## Abstract

**Background:**

Even after accounting for deprivation, mortality rates are higher in Scotland relative to the rest of Western Europe. Higher mortality from alcohol- and drug-related deaths (DRDs), violence and suicide (particularly in young adults) contribute to this ‘excess’ mortality. Age-period and cohort effects help explain the trends in alcohol-related deaths and suicide, respectively. This study investigated whether age, period or cohort effects might explain recent trends in DRDs in Scotland and relate to exposure to the changing political context from the 1980s.

**Methods:**

We analysed data on DRDs from 1979 to 2013 by sex and deprivation using shaded contour plots and intrinsic estimator regression modelling to identify and quantify relative age, period and cohort effects.

**Results:**

The peak age for DRDs fell around 1990, especially for males as rates increased for those aged 18 to 45 years. There was evidence of a cohort effect, especially among males living in the most deprived areas; those born between 1960 and 1980 had an increased risk of DRD, highest for those born 1970 to 1975. The cohort effect started around a decade earlier in the most deprived areas compared to the rest of the population.

**Conclusion:**

Age-standardised rates for DRDs among young adults rose during the 1990s in Scotland due to an increased risk of DRD for the cohort born between 1960 and 1980, especially for males living in the most deprived areas. This cohort effect is consistent with the hypothesis that exposure to the changing social, economic and political contexts of the 1980s created a delayed negative health impact.

**Electronic supplementary material:**

The online version of this article (10.1186/s12889-018-5267-2) contains supplementary material, which is available to authorized users.

## Background

Since around 1950 Scotland has had higher, and more slowly improving, mortality rates than the rest of Western Europe [1–3]. From 1981 onwards less and less of the higher mortality in Scotland compared to England and Wales could be explained by deprivation [4, 5]. Higher mortality from alcohol- and drug-related deaths (DRDs), violence and suicide (particularly in young adults) and higher mortality from heart disease, stroke and cancer throughout adulthood contribute to this ‘excess’ mortality (i.e. higher mortality over and above that explained by differences in socioeconomic deprivation), the slower improvement in mortality compared with the rest of Europe and the greater health inequalities [6, 7]. It has been suggested that a third of Scotland’s ‘excess’ mortality over England among 15 to 54 year olds can be accounted for by a higher prevalence of problem drug use in Scotland, which rose rapidly in Scotland in the 1980s [8].

It has been proposed that these mortality phenomena are best explained in terms of the interaction of the neoliberal political and economic policies of the 1980s (which favoured monetarist economic policies and led to a rapid rise in income inequality and an increase in unemployment) with the underlying vulnerabilities in the Scottish population due to historical regional and urban policy (which meant that the population in Scotland had different housing, employment and social context, which was more badly affected) [6, 7, 9, 10]. These policy changes may have impacted on health in the same way as in other countries [11–13]. Part of the Scottish population, particularly the working class living in the deindustrialising regions, may have been particularly badly affected by these dramatic changes of the 1980s [6, 10, 11, 14–17].

If this impact of political change was partially responsible for the mortality phenomena in Scotland, then the worst affected cohort of individuals would be those most directly exposed (i.e. working class, young adults first experiencing working life during 1979–1990). While more males would have been impacted directly in terms of job losses, females would have been affected by the loss of household income and the associated effect of the changing political context. There is, however, ambiguity around this exposure cohort; the timing of the exposure is rather indistinct with deindustrialisation occurring earlier [18] and the change in political approach persisted after [11, 12].

Period or cohort effects (age effects would have been adjusted for in the age-standardised analyses) may be partially responsible for the trends in DRDs in Scotland, something already identified for alcohol-related mortality and suicide [19, 20]. DRDs have continued to increase in Scotland but with large differences between groups [21, 22]. For instance, males account for the majority of deaths and the average age of those dying has increased reflecting an ageing profile of problem drug users (problem drug use is defined by Information Services Division Scotland (ISD) Scotland as the problematic use of opiates (including illicit and prescribed methadone use) and/or the illicit use of benzodiazepines and implies routine and prolonged use as opposed to recreational and occasional drug use) [23]. Consistent with this, the prevalence of drug use has fallen among adolescents, although the vast majority of the reported drug use at this age relates to drugs that do not carry a high risk of mortality [24, 25]. There are also clear differences in DRD rates by deprivation, with higher rates for those living in more deprived areas [22, 26].

In this paper we explore whether there are age, period or cohort (APC) effects in DRDs in Scotland corresponding to the changing political context from the 1980s, which might help explain these recent trends in DRDs in Scotland.

## Methods

### Data

We obtained data on the number of DRDs by sex, single year of age at death and year of registration of death for Scotland from 1979 to 2013, and mid-year population estimates for Scotland by sex and single year of age, from age 0 to 89 years (with pooled data for those aged 90 years and over), from 1979 to 2013, from the National Records of Scotland (NRS).

DRDs from 1979 to 1999 were defined by the International Classification of Diseases, Ninth Revision 9 (ICD9) codes 292, 304, 305.2–305.9, E850-E858, E950.0-E950.5, E962.0, and E980.0-E980.5 and from 2000 onward by the ICD10 codes F11-F16, F18–19, X40-X44, X60-X64, X85, and Y10-Y14. This follows the Office for National Statistics’ ‘wide’ definition for deaths from drug poisoning, referred to in this paper as DRDs, which include both deaths from poisoning from any drug or medication as well as controlled drugs (see NRS [27] for a discussion of data availability including why only data on DRDs post-1979 are used and Additional file 1: Figure S1 to compare DRDs determined by other common definitions).

Analyses by deprivation were based on Carstairs deprivation categories and compared the most deprived quintile to the remaining quintiles grouped to create the less deprived four-fifths of the population (this allowed sufficient numbers for analysis) [28]. For DRDs data, Carstairs look-up files were obtained from the ISD website; death data were matched to Carstairs quintiles using the appropriate output area (mean population c.115 in 2011). Data were then assigned to an appropriate Carstairs time period; cases with no associated Carstairs (2.4% of dataset) were removed from the analyses by deprivation. Population data by Carstairs, from age 0 to 84 years (with pooled data for those aged 85 years and over), were obtained from ISD with interpolation between the censuses.

Unless stated otherwise, analyses were restricted to those aged 15–89 years, or aged 15–84 years for deprivation analysis.

### Descriptive analysis - rates

For presentation of trends, death rates for all ages from 1979 to 2013 by sex were age-standardised to the 2013 version of the European Standard Population. For age effects analysis, data from 1979 to 1996 and data from 1997 to 2013 were separately combined for presentation of crude death rates by five-year age groups (from 0 to 4 years to 90+ years (85+ years for deprivation analysis)) for age at death by sex.

To examine APC effects, DRD rates were plotted using data grouped by age at death (five-year age groups), year of death (five-year periods) and birth cohort (those born over five-year periods, estimated from age at death and year of registration of death. Additional file 1: Figure S2 shows the cohorts, and their corresponding age at death, covered by our dataset).

### Descriptive analysis - age-year and age-cohort shaded contour plots

Shaded contour plots (SCPs) were produced in R version 3.3.0 (in these Lexis surface visualisations, lines and colour show population age groups at particular points in time with the same DRD rates) [29]. Separate plots were produced by sex, and then separately by deprivation, created using rates for single age and year of death or for single age and birth year (for information on SCPs see Appendix 1 in Parkinson et al. (2016) [20]). In age-year SCPs, where age is on the vertical axis and year on the horizontal axis, period effects appear as vertical patterns, age effects as horizontal patterns and cohort effects as diagonal ‘disruptions’ running along diagonal lines from the bottom left to top right. In age-cohort SCPs the data are rearranged by birth cohort on the horizontal axis so cohorts instead run vertically from bottom to top of the figure and period effects appear as diagonal lines running from bottom right to top left.

### Inferential analysis – Intrinsic estimator models

For APC regression modelling, DRD and population data were grouped into five-year age groups and five-year time periods stratified by sex and deprivation. The year for the cohorts was calculated as a function of the five-year age and period groups, as being equal to mean period minus mean age. Standard negative binomial regression models were initially run to ensure that age, period or cohort alone or any combination of two factors did not fit the data better than a full APC model. The intrinsic estimator (IE) command (apc_ie) in Stata version 13 (Stata Corp LP, College Station, Texas, USA) was used [30]. As the count data were over-dispersed, negative binomial models were used. Models were fitted separately for each sex and deprivation specific strata. For the most deprived fifth of females, the 1899 cohort data were replaced by those of the 1904 cohort to obtain a suitable model. This equated to replacing data for age group 80–84 years and period 1979–1983 with that of age group 80–84 years and period 1984–1988. The replacement was made at sub-group level, which would not have significantly affected trends reported.

## Results

### Age-sex-standardised trends over time

From 1979 until 1986, age-standardised DRD rates (Additional file 1: Figure S3) for both males and females were low and decreased following similar trends and levels. Subsequently, rates rose for both sexes but much faster for males, reaching a level in 2013 far greater than that of 1979, while those for females increased to a level similar to that of 1979.

These worsening trends in DRDs were sharpest for those living in the most deprived areas and for males (Additional file 1: Figure S4). Indeed, for those living in less deprived areas, rates for males increased slowly only after 1991 and those for females remained consistently low.

### Age-year and age-cohort shaded contour plots

Rates for DRDs differed markedly over the life-course (Additional file 1: Figures S5 and S6) and the age profiles have changed over the period and differ by sex. The peak rate for females was initially older than that for males, but this fell as rates decreased around 1985 for females aged 50–60 years and then increased around the mid-1990s for young adult females aged 20–30 years resulting in the peak age becoming more comparable to that for males.

**Fig. 1:**
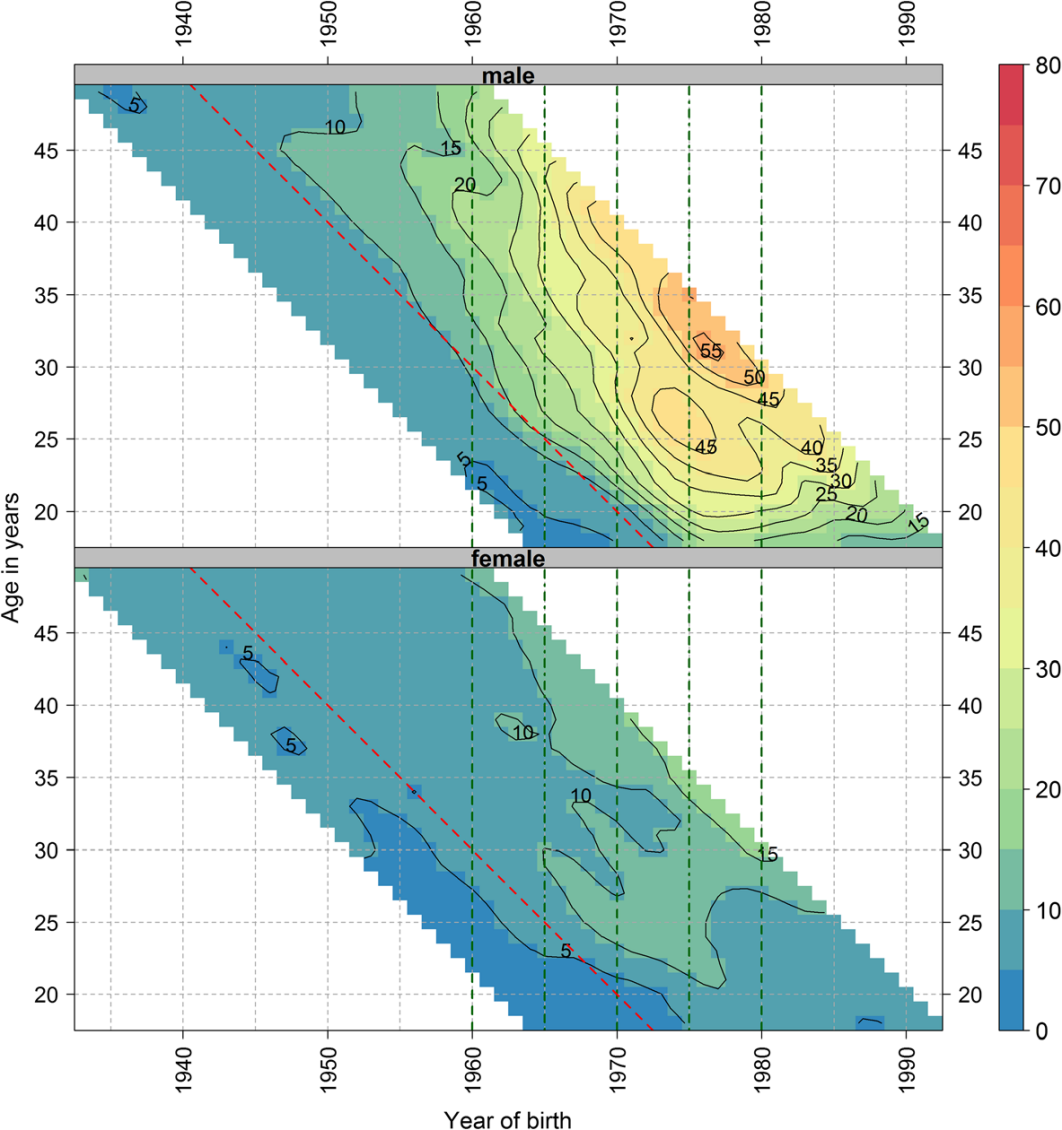
Smoothed shaded contour plot of age-specific crude drug-related death rates per 100,000 population in Scotland for single age and birth year by sex*. * Single age is from 18 to 49 years and birth year from 1933 to 1992. The colour and contour labels indicate the number of drug-related deaths per 100,000 for each single age and year. Green vertical dashed guidelines indicate birth cohorts 1960 (left most line), 1970 and 1980 (right most line) and dashed-dotted guidelines mid-decadal birth cohorts 1965 and 1975. Red diagonal dashed guideline indicates calendar year 1990. The three most recent and three oldest years and ages have been removed from the figure to avoid giving the impression trends are falling at the end due to an artefact of smoothing

Around 1990, the peak age of DRDs fell for males as rates increased for those aged around 18 to 45 years, and especially for those < 30 years. Subsequently the peak age began to increase, suggesting a cohort effect with the most affected cohort born between 1970 and 1980, where DRDs peaked at about 45/100,000 for 25 year olds (around the 1975 cohort) and also 5 years later at 65/100,000 for 30 year olds (Fig. 1). This effect was obvious after calendar year 1990 (red line in Fig. 1). A similar but weaker cohort effect was seen in females born between 1965 and 1980.

**Fig. 2:**
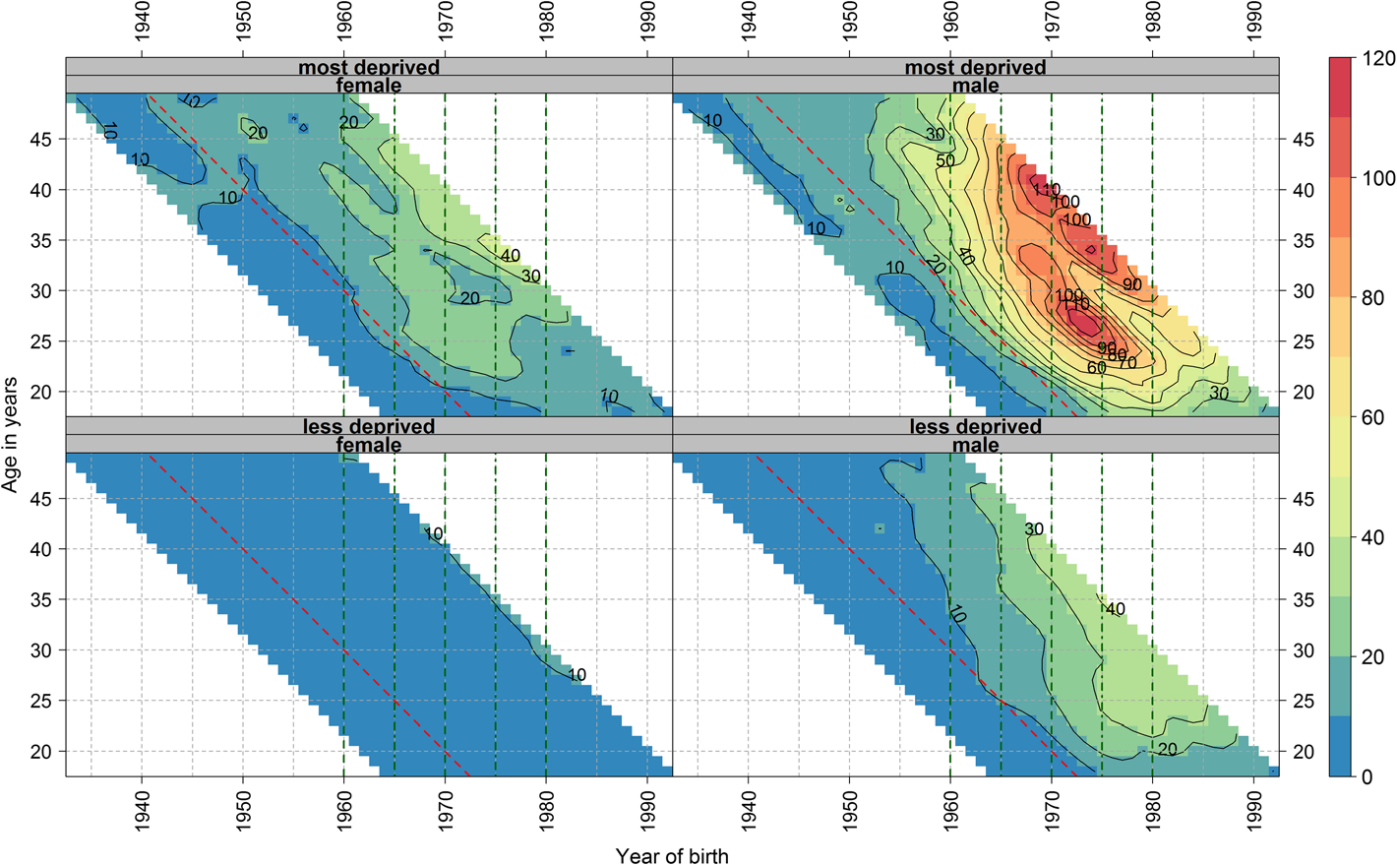
Smoothed shaded contour plot of age-specific crude drug-related death rates per 100,000 population in Scotland for single age and birth year stratified by sex and deprivation*. * Single age is from 18 to 49 years and birth year from 1933 to 1992. Green vertical dashed guidelines indicate birth cohorts 1960 (left most line), 1970 and 1980 (right most line) and dashed-dotted guidelines mid-decadal birth cohorts 1965 and 1975. Red diagonal dashed guideline indicates calendar year 1990. The three most recent and three oldest years and ages have been removed from the figure to avoid giving the impression trends are falling at the end due to an artefact of smoothing

The age profiles by deprivation (Additional file 1: Figures S7 and S8) showed clear differences by deprivation and over time for both males and females. The vertical pattern in Fig. 2 indicates a cohort effect of higher DRDs for those born between around 1960 and 1980, with men and those in deprived areas affected most. This effect was again obvious after calendar year 1990 (red line in Fig. 2). The cohort effect also began up to a decade earlier for males in the most deprived group compared to the less deprived group. For men in the most deprived group, the most affected cohort, born between around 1970 and 1975, experienced high DRD risk at two stages in their life course, both in their mid to late twenties and then in their mid to late thirties.

### Period analysis

Analysis by period showed that the peak age for DRDs for males decreased from 1979-1983 to 1994–1998 before increasing again as the cohort born in the early 1970s aged, appearing to carry with them an increased DRD risk compared to previous cohorts (Additional file 1: Figure S9). The effect was less clear for females.

**Fig. 3:**
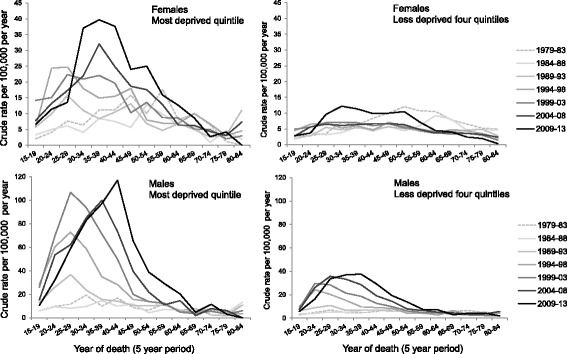
Crude drug-related death rates per 100,000 population per year in Scotland by age, period, sex and deprivation*. * age is by five-year age groups for ages 15–84 years and period by five-year periods 1979–83 to 2009–13

The cohort effect was clear for males living in both deprivation groups, with higher rates for those in the most deprived areas (Fig. 3). Rates also started to increase five years earlier for the most deprived group resulting in the peak age group for each period being five years older for this deprivation group. A weaker cohort effect was also evident for females living in the most deprived areas. The overall DRD trends are largely accounted for by the trends in the most deprived population fifth.

### IE analysis

**Fig. 4:**
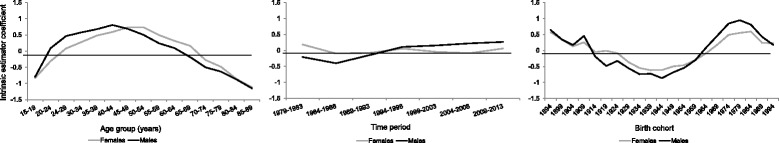
Intrinsic estimator coefficients for age, period and birth cohort effects for drug-related deaths in Scotland stratified by sex

**Fig. 5:**
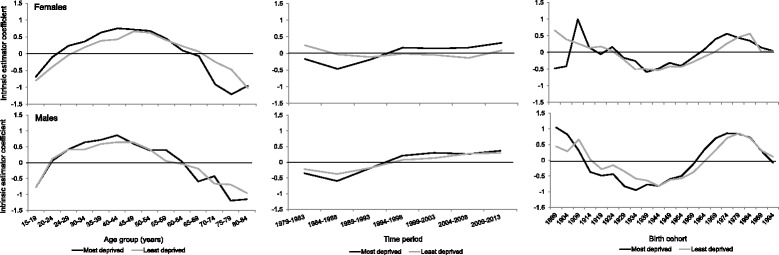
Intrinsic estimator coefficients for age, period and birth cohort effects for drug-related deaths in Scotland stratified by sex and deprivation*. * The data for the oldest birth cohort in females in the most deprived group was substituted with that for the 1904 cohort

Regression analysis confirmed that no single or two factor combination of age, period or cohort provided a better fit than the full APC model (Additional file 2). Results from the APC models showed congruence between the plots of IE coefficients (Figs. 4 and 5) and the SCPs (Figs. 1 and 2).

The IE coefficients (see Additional file 2 for how to interpret IE coefficients) suggested that there is a strong age effect for both sexes, with a peak in middle age (younger and larger among males); a small period effect for males, increasing through the 1990s, but little effect for females; and a birth cohort effect for both sexes, with a low around the 1944 cohort (Fig. 4) (see Additional file 3 for the IE coefficient data with 95% confidence intervals). A larger cohort effect was observed for males than females in the successive birth cohorts 1959 to 1989.

The deprivation stratified data showed similar overall patterns to the whole population for males (Fig. 5) (see Additional file 3 for the IE coefficient data with 95% confidence intervals). The timing of the cohort effects differed between deprivation groups, with the effect since the 1960s increasing five years earlier in the most deprived population. For females, both deprivation populations showed age and cohort effects, similar to those for males, but with slightly different timings, while a period effect similar to that for males was only evident for the most deprived group. As for males, the cohort effect since the 1960s increased earlier in the most deprived population.

## Discussion

### Main results

Between 1979 and 1986 the DRD rate in Scotland decreased and was similar for males and females. Subsequently, rates for men increased, due to a sharp rise among males living in the most deprived areas. In contrast, rates for females increased more slowly, also primarily due to a rise among females in the most deprived areas, leading to a growing gap between the sexes.

Around 1990, DRD rates increased noticeably (particularly for young males and in the most deprived areas), and the cohort born between around 1960 and 1980 experienced considerably higher DRD rates until the end of the time series and are primarily responsible for the increasing number of deaths over time. A similar although weaker cohort effect was observed for females. The cohort effect was more prominent for those living in the most deprived areas for both sexes, where it also started around a decade earlier than for the rest of the population. The IE analyses supported the descriptive findings of a cohort effect alongside the known age patterning and suspected period effect from the 1990s.

### Strengths and weaknesses of the analysis

Defining a DRD is not straightforward and changes over the years make establishing trends problematic [21]. To obtain the longest time trend, we used the ONS ‘wide’ definition rather than the ‘baseline’ definition for the UK Drugs Strategy used to report DRDs by NRS and many other statistical bodies in Scotland [21, 27]. The method for this ‘wide’ definition has not changed over our time series, but its use means that figures for DRDs are higher than those obtained using other definitions, as it includes both deaths from poisoning from any drug or medication as well as controlled drugs. However, long-term trends (if not the levels) are broadly the same using the different definitions (Additional file 1: Figure S1).

Although NRS has information on each death registered in Scotland since 1974, data on DRDs were restricted to post-1979 when NRS started to use ICD9 codes; ICD8 codes were used from 1974 to 1978 but there is no suitable cross-match between codes that may be used for DRDs [27]. The numbers identified by ICD9 codes are considered to give a good indication of what happened between 1979 and 1999 [27]. Thereafter, ICD10 codes have been used. There is some overlap in the ICD codes used to identify DRDs and suicides. These codes concern deaths due to intentional self-poisoning or undetermined intent poisoning by drugs, medicaments and biological substances. The number of DRDs that are a subset of suicides is greater for ICD9 than ICD10 coding and also for the ONS definition than for the NRS used definition, due to the way the data are obtained.

We used data with the year the death was registered rather than the year it occurred (chosen to match the reporting convention of NRS) but the impact on our conclusions is negligible [21]. The disaggregation by age over time led to small numbers in each group and a substantial degree of random variation in the Lexis surface but the confidence intervals for the IE analyses and the rounding in the SCPs ensure that the patterns are very unlikely to be due to chance.

Modelling APC effects simultaneously to disentangle their effects is problematic due to their mathematical co-dependence, the ‘identification problem’. There is disagreement as to whether this is possible [31–34] and proposed methods to separate APC effects have limitations [35–37]. Imposing a constraint on the model, such as applying an assumption of equality between groups to break the co-dependence, is a common solution but the results are sensitive to the chosen assumptions and there is often little basis for making these [38]. The IE approach used here assumes that the sum of the coefficients in the vector are zero thus avoiding the need to make such constraints; although it is not without limitations [30], it is used here to provide an inductive exploration of the hypothesis to complement the line plots and SCPs. This triangulation adds strength to the findings; there is congruence between the IE plots and SCPs.

Finally, we only have a partial view of each birth cohort as we do not have data for all years for all cohorts. This is always the case with APC analyses and it is mitigated through the comparison of age-specific rates for different cohorts and time periods. We can, however, only have confidence that our observations are valid for the period 1979–2013.

### Comparison with other studies

Methodological differences in recording of DRDs make comparison of DRDs between countries problematic. However, a recent comparison indicates that the Scottish drug-death rate could be at least double that of the UK as a whole [21]. The trends in Scottish DRDs over time and differences between males and females and between age groups have been previously described for Scotland [21]. The substantially higher rates of DRDs among those living in deprived circumstances is also well-recognised as is the increase in the average age of individuals dying from DRDs from 1996 to 2014, seen as reflecting an ageing profile of problem drug users and evidenced here as a cohort effect [21, 22, 26].

The findings presented here echo those of recent APC research on suicide in Scotland where a cohort effect was identified for people born between 1960 and 1980 [20]. Both cohort effects are largely driven by males and those living in the most deprived areas but the cohort most affected by suicide occurs slightly earlier than that for DRDs, 1965–1974 versus 1970–1980, respectively. Further, although for both health outcomes the timing of the cohort effect and cohort at highest risk differs by deprivation, being earlier for males in the most deprived group, this difference is not the same. For suicides there is around a five year difference while for DRDs it is around ten years. There are also similarities to the finding of an age-period interaction for alcohol-related deaths in Scotland which impacted on the same birth cohorts as those most affected by suicide and DRDs [19]. These APC results are consistent with these health outcomes being important in explaining a growing relative mortality risk for young to middle-aged males since the mid-1990s [39].

Recently Walsh et al. [7] showed that ‘excess’ mortality in Scotland can be best explained by a greater vulnerability, due to historical processes of urban change and selective migration to new towns, to economic and social changes resulting from the political changes of the 1980s. The cohort effect identified here is consistent with exposure of young working-age adults to the changed socioeconomic context during the 1980s, an event which would have occurred primarily when young adults entered the job market and therefore earlier for those in the most deprived areas, and adds further evidence to this thesis. It seems likely that alcohol-related deaths, DRDs and suicide outcomes that contribute to Scotland’s ‘excess’ mortality could share a common causal pathway stemming from the changing social and economic policies of the 1980s. It has also been suggested recently that there is a similar patterning of external causes of death for particular cohorts in the USA [40]. Another possibility is that there was a change in the type of drugs available for the cohorts with the highest DRDs as they moved into adulthood, as hard drugs became more available. However, we would have expected any change in DRDs due to the introduction of new drugs to society to have also been evident in subsequent cohorts.

## Conclusions

DRDs negatively impact society [41, 42] and make an important contribution to the health inequalities and ‘excess’ mortality in Scotland [6]. The rationale for our study was to investigate whether there is evidence of a cohort effect for those most likely to have been exposed to the negative consequences of the changing social, economic and political contexts of the 1980s. The cohort effect we identify here for DRDs, which appears for young adult males (and to a lesser extent for females) in the early 1990s, seems consistent with this hypothesis given that young adults would have entered the job market at this time and, particularly in the most deprived areas, been exposed to high unemployment levels and reduced support [43, 44].

The full impact of ‘excess’ mortality, and indeed morbidity, in cohorts with high DRDs is unlikely to be known for some time. As the cohort of people at greatest risk of DRDs continues to age, drugs services will need to adapt to their needs as co-morbidities from chronic conditions associated with ageing and drug use become more prevalent. There is also a risk that more recent exposures to a more ‘flexible’ labour market and greater conditionality and sanctions in the social security system, particularly for young working-age adults, may in time lead to another cohort at high risk [45, 46]. Continued surveillance of this population is therefore merited.

## Additional files

Additional file 1: **Figure S1.** Drug-related deaths in Scotland as determined by different definitions. **Figure S2.** Age and period ranges for birth cohorts available in the drug-related deaths dataset, 1979–2013. **Figure S3.** European age-standardised drug-related death rates per 100,000 population per year in Scotland, 1979–2013, by sex. **Figure S4.** European age-standardised drug-related death rates per 100,000 population per year in Scotland, 1979–2013, by sex and deprivation. **Figure S5.** Age distribution of crude drug-related death rates per 100,000 population per year in Scotland over periods 1979–1996 and 1997–2013 combined data by sex. **Figure S6.** Smoothed shaded contour plot of age-specific crude drug-related death rates per 100,000 population in Scotland for each single age from 18 to 86 years of age and each year from 1982 to 2010 stratified by sex. **Figure S7.** Age distribution of crude drug-related death rates per 100,000 population per year in Scotland over periods 1979–1996 and 1997–2013 combined data by sex and deprivation. **Figure S8.** Smoothed shaded contour plot of age-specific crude drug-related death rates per 100,000 population in Scotland for each single age from 18 to 81 years of age and each year from 1982 to 2010 stratified by sex and deprivation. **Figure S9.** Crude drug-related death rates per 100,000 population per year in Scotland by age (five-year age groups, for ages 15–89 years), period (five-year periods 1979–83 to 2009–13) and sex. (PDF 384 kb)

Additional file 2: Regression models and interpretation of intrinsic estimator coefficients. **Table S1.** Goodness-of-fit statistics for single and two factor regression models and APC model. **Table S2.** Goodness-of-fit statistics for single and two factor regression models and APC model by Carstairs deprivation category. (PDF 305 kb)

Additional file 3: Intrinsic estimator statistics. **Table S1.** Intrinsic estimator coefficient statistics for age, period and birth cohorts for drug-related deaths in Scotland for females. **Table S2.** Intrinsic estimator coefficient statistics for age, period and birth cohorts for drug-related deaths in Scotland for males. **Figure S1.** Intrinsic estimator coefficients with 95% confidence intervals for age, period and birth cohorts for drug-related deaths in Scotland stratified by sex. **Table S3.** Intrinsic estimator coefficient statistics for age, period and birth cohorts for drug-related deaths in Scotland for females living in the most deprived quintile. **Table S4.** Intrinsic estimator coefficient statistics for age, period and birth cohorts for drug-related deaths in Scotland for females living in the less deprived four quintiles. **Table S5.** Intrinsic estimator coefficient statistics for age, period and birth cohorts for drug-related deaths in Scotland for males living in the most deprived quintile. **Table S6.** Intrinsic estimator coefficient statistics for age, period and birth cohorts for drug-related deaths in Scotland for males living in the less deprived four quintiles. **Figure S2.** Intrinsic estimator coefficients with 95% confidence intervals for age, period and birth cohorts for drug-related deaths in Scotland for females stratified by deprivation. **Figure S3.** Intrinsic estimator coefficients with 95% confidence intervals for age, period and birth cohorts for drug-related deaths in Scotland for males stratified by deprivation. (PDF 1151 kb)

## Abbreviations

APC: age-period-cohort
DRD: drug-related death
ICD: International Classification of Diseases
IE: intrinsic estimator
ISD: Information Services Division Scotland
NRS: National Records of Scotland
SCP: shaded contour plot.
