# Supplementary material for: Drug-related deaths in Scotland 1979–2013: evidence of a vulnerable cohort of young men living in deprived areas
Source: BMC Public Health. 2018 Mar 27;18:357. doi: 10.1186/s12889-018-5267-2 (PMC5870372; doi:10.1186/s12889-018-5267-2)

Additional file 1: Supporting figures

Figure S1 Drug-related deaths in Scotland as determined by different definitions

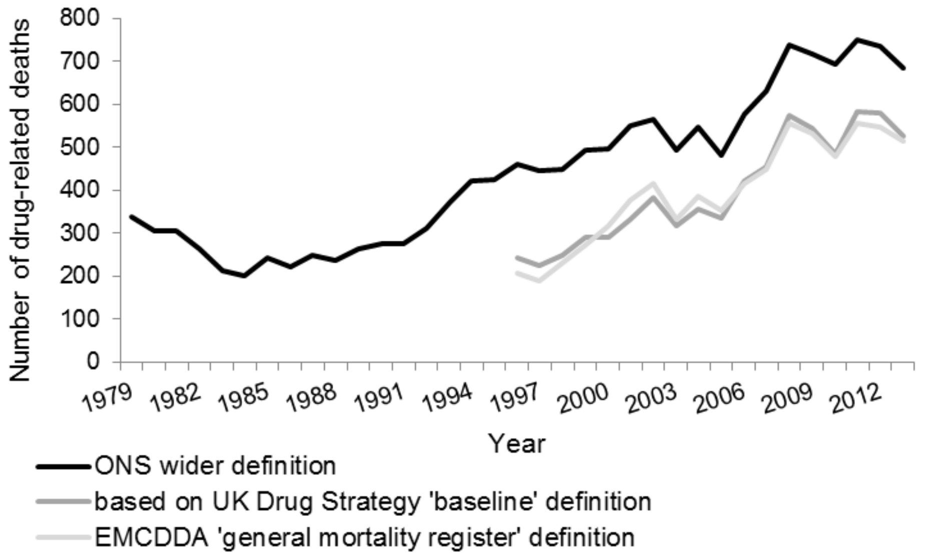

EMCDDA = European Monitoring Centre for Drugs & Drug Addiction
The ONS wide definition counts deaths from poisoning by any drug or medicament, including (for example) paracetamol and anti-depressants, as well as controlled drugs like heroin and cocaine

Figure S2 Age and period ranges for birth cohorts available in the drug-related deaths dataset, 1979-2013

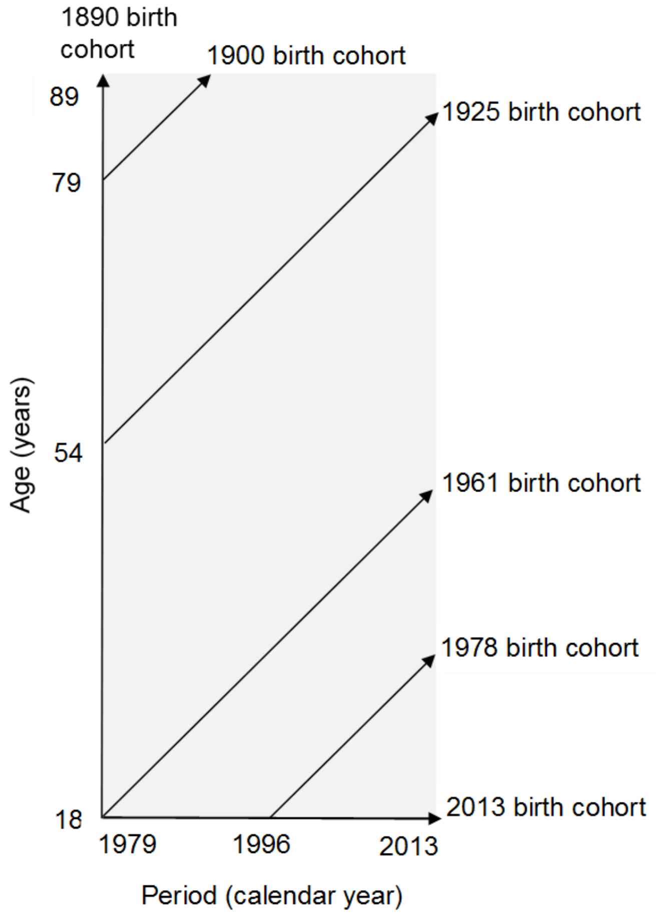

Figure S3 European age-standardised drug-related death rates per 100,000 population per year in Scotland, 1979-2013, by sex

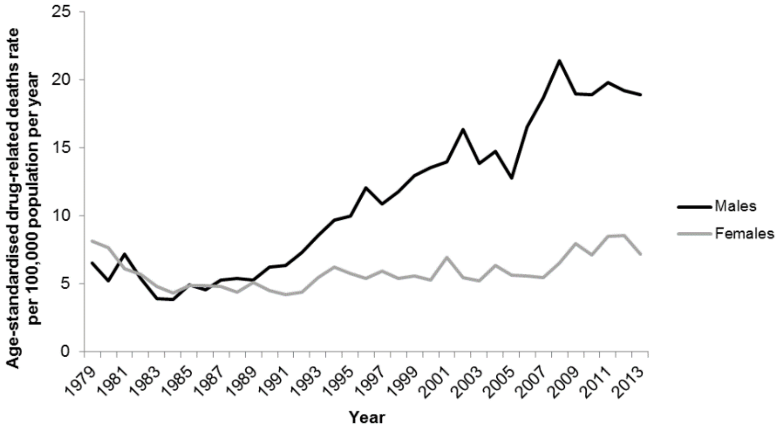

Figure S4 European age-standardised drug-related death rates per 100,000 population per year in Scotland, 1979-2013, by sex and deprivation

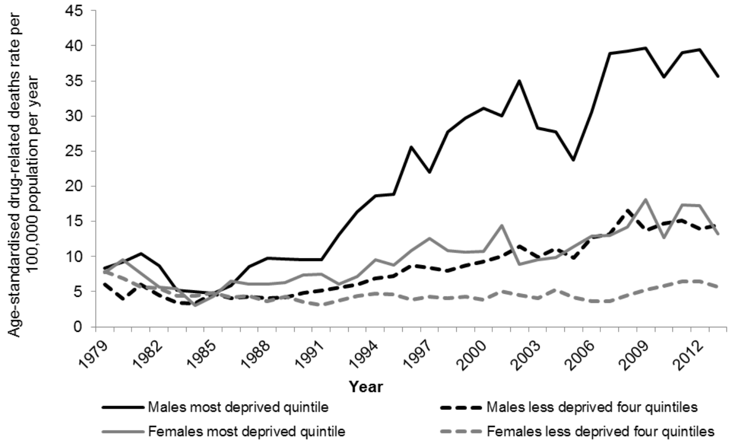

Figure S5 Age distribution of crude drug-related death rates per 100,000 population per year in Scotland over periods 1979-1996 and 1997-2013 combined data by sex*

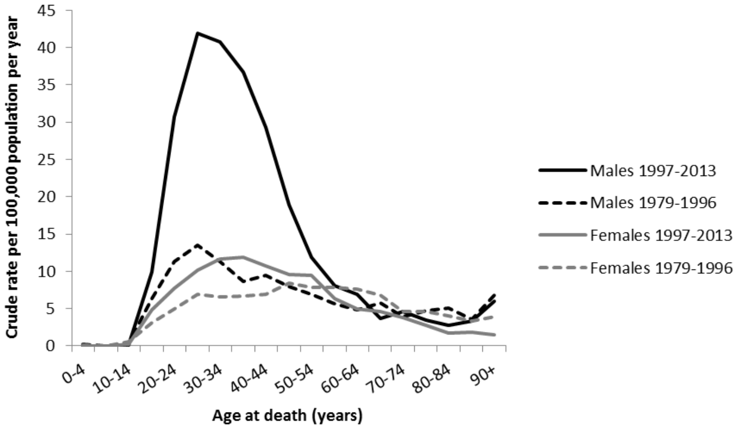

* These age profiles show general trends with age across two time periods 1979-1996 and 1997-2013 and will have been affected by any age-period-cohort effects occurring during those periods.

Figure S6 Smooth shaded contour plot of age-specific crude drug-related death rates per 100,000 population in Scotland for each single age from 18 to 86 years of age and each year from 1982 to 2010 stratified by sex*

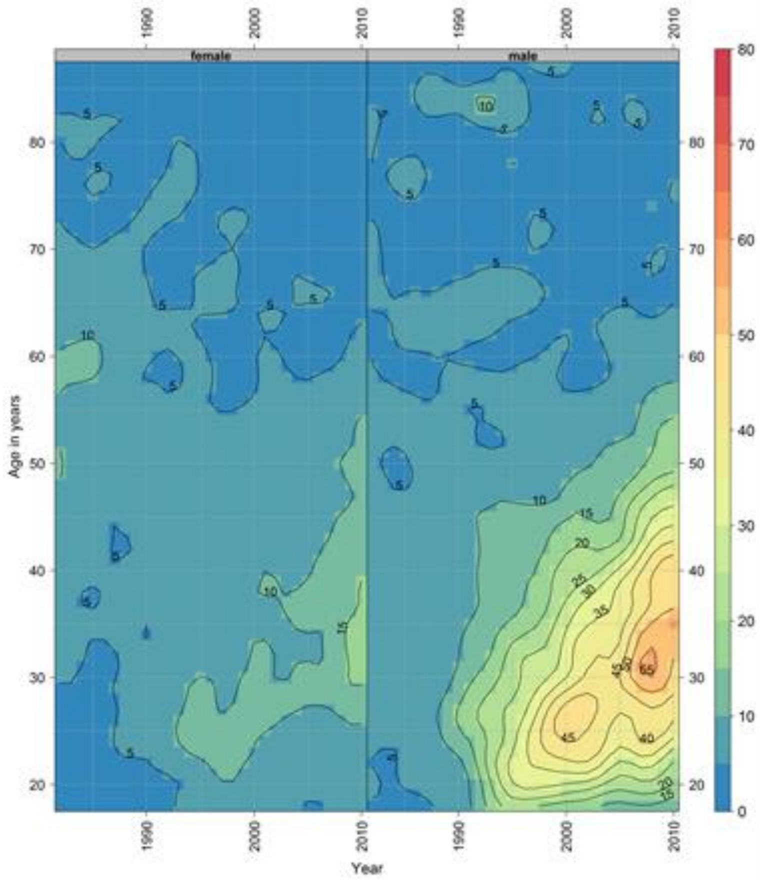

***** The colour and contour labels indicate the number of drug-related deaths per 100,000 for each single age and year. The three most recent and three oldest years and ages have been removed from the figure to avoid giving the impression trends are falling at the end due to an artefact of smoothing.

Figure S7 Age distribution of crude drug-related death rates per 100,000 population per year in Scotland over periods 1979-1996 and 1997-2013 combined data by sex and deprivation*

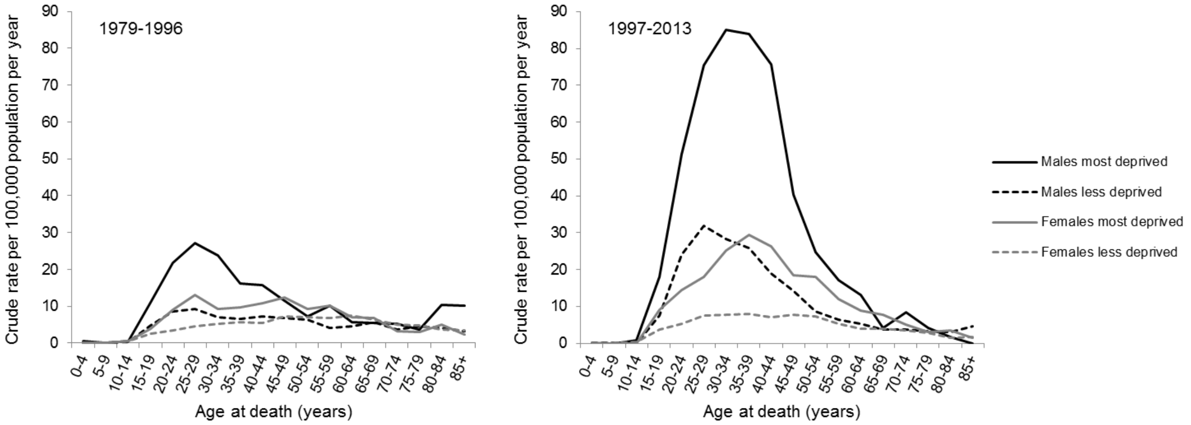

* These age profiles show general trends with age across two time periods 1979-1996 and 1997-2013 and will have been affected by any age-period-cohort effects occurring during those periods.

Figure S8 Smooth shaded contour plot of age-specific crude drug-related death rates per 100,000 population in Scotland for each single age from 18 to 81 years of age and each year from 1982 to 2010 stratified by sex and deprivation (most deprived fifth and less deprived four-fifths)*

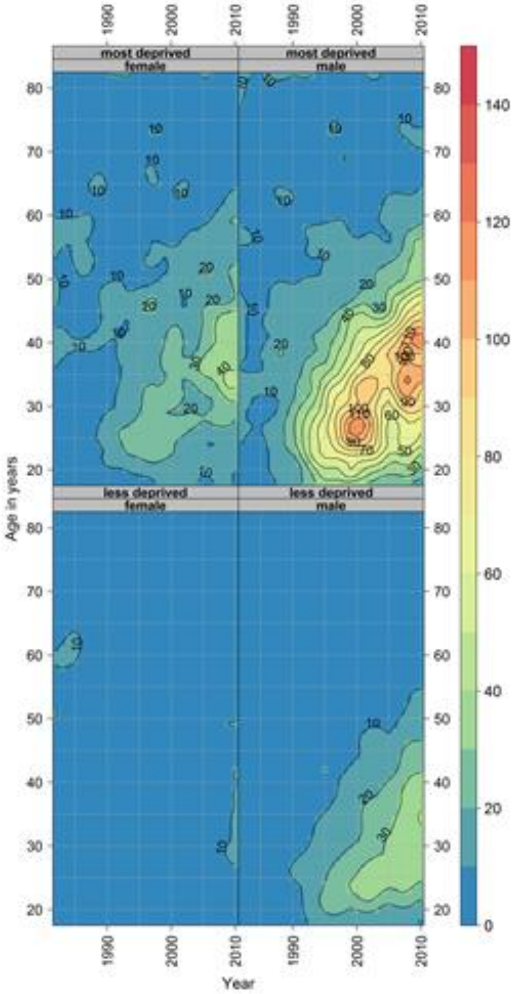

***** The colour and contour labels indicate the number of drug-related deaths per 100,000 for each single age and year. The three most recent and three oldest years and ages have been removed from the figure to avoid giving the impression trends are falling at the end due to an artefact of smoothing.

Figure S9 Crude drug-related death rates per 100,000 population per year in Scotland by age (five-year age groups, for ages 15-89 years), period (five-year periods 1979-83 to 2009-13) and sex

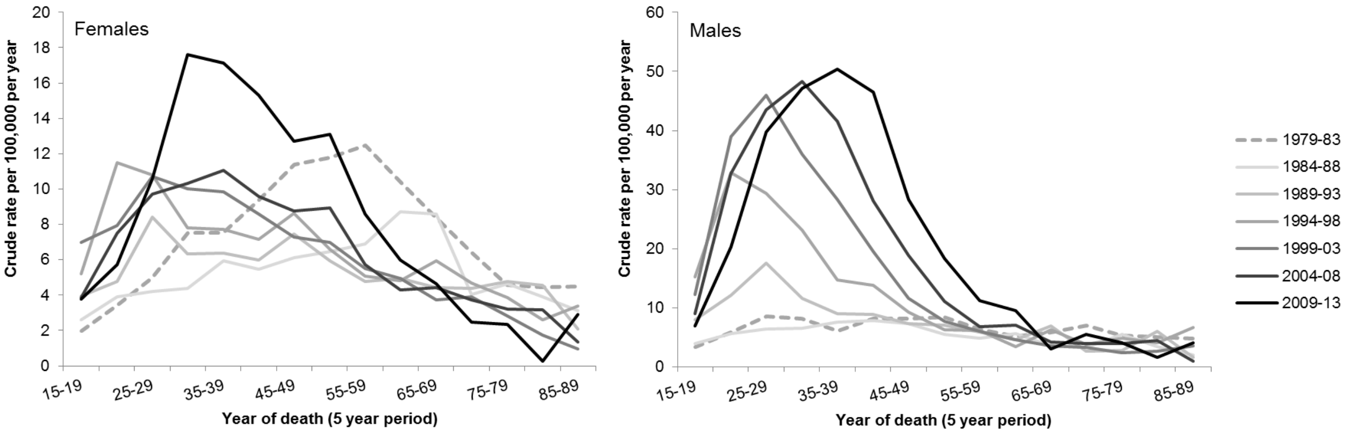

Supplement: Supplementary file 1 — Figure S1. Drug-related deaths in Scotland as determined by different definitions. Figure S2. Age and period ranges for birth cohorts available in the drug-related deaths dataset, 1979–2013. Figure S3. European age-standardised drug-related death rates per 100,000 population per year in Scotland, 1979–2013, by sex. Figure S4. European age-standardised drug-related death rates per 100,000 population per year in Scotland, 1979–2013, by sex and deprivation. Figure S5. Age distribution of crude drug-related death rates per 100,000 population per year in Scotland over periods 1979–1996 and 1997–2013 combined data by sex. Figure S6. Smoothed shaded contour plot of age-specific crude drug-related death rates per 100,000 population in Scotland for each single age from 18 to 86 years of age and each year from 1982 to 2010 stratified by sex. Figure S7. Age distribution of crude drug-related death rates per 100,000 population per year in Scotland over periods 1979–1996 and 1997–2013 combined data by sex and deprivation. Figure S8. Smoothed shaded contour plot of age-specific crude drug-related death rates per 100,000 population in Scotland for each single age from 18 to 81 years of age and each year from 1982 to 2010 stratified by sex and deprivation. Figure S9. Crude drug-related death rates per 100,000 population per year in Scotland by age (five-year age groups, for ages 15–89 years), period (five-year periods 1979–83 to 2009–13) and sex. (PDF 384 kb) [file 12889_2018_5267_MOESM1_ESM.pdf]
