# Supplementary material for: Drug-related deaths in Scotland 1979–2013: evidence of a vulnerable cohort of young men living in deprived areas
Source: BMC Public Health. 2018 Mar 27;18:357. doi: 10.1186/s12889-018-5267-2 (PMC5870372; doi:10.1186/s12889-018-5267-2)
Supplement: Supplementary file 2 — Regression models and interpretation of intrinsic estimator coefficients. Table S1. Goodness-of-fit statistics for single and two factor regression models and APC model. Table S2. Goodness-of-fit statistics for single and two factor regression models and APC model by Carstairs deprivation category. (PDF 305 kb) [file 12889_2018_5267_MOESM2_ESM.pdf]

Additional file 2: Regression models and interpretation of intrinsic estimator coefficients

Regression models

The goodness-of-fit statistics for the single and two factor regression models and APC model are shown in Table 1. The IE model with the lowest AIC and the highest log-likelihood values was the best model when compared with the other simple models.

Table 1 Goodness-of-fit statistics for single and two factor regression models and APC model

Model	Female				Male			
	df	Log-likelihood	AIC	r^2	df	Log-likelihood	AIC	r^2
Age	16	-423	879	0.09	16	-486	1003	0.11
Period	8	-461	938	0.01	8	-525	1065	0.03
Cohort	22	-449	942	0.04	22	-462	968	0.15
Age+period	22	-416	877	0.11	22	-461	967	0.15
Age+cohort	36	-362	796	0.22	36	-382	837	0.29
Period+cohort	28	-440	936	0.05	28	-458	972	0.16
Age+period+cohort (intrisic estimator)	40	-346	772	n/a	40	-367	816	n/a

df, degrees of freedom; AIC, Akaike information criterion

The goodness-of-fit statistics for the single and two factor regression models and APC model by Carstairs deprivation group are shown in Table 2. The IE model with the lowest AIC and the highest log-likelihood values was the best model when compared with the other simple models.

Table 2 Goodness-of-fit statistics for single and two factor regression models and APC model by Carstairs deprivation category

Model	Female - More deprived*				Female - Less deprived			
	df	Log-likelihood	AIC	r^2	df	Log-likelihood	AIC	r^2
Age	15	-323	675	0.11	15	-365	759	0.07
Period	8	-348	712	0.04	8	-385	785	0.02
Cohort	20	-327	694	0.09	21	-385	813	0.02
Age+period	21	-299	641	0.17	21	-355	752	0.09
Age+cohort	34	-271	610	0.25	34	-311	690	0.21
Period+cohort	27	-320	691	0.11	27	-375	804	0.04
Age+period+cohort (intrisic estimator)	38	-260	596	n/a	38	-291	659	n/a
**Model**	**Male - More Deprived**				**Male - Less Deprived**			
	df	Log-likelihood	AIC	r^2	df	Log-likelihood	AIC	r^2
Age	15	-382	794	0.11	15	-427	885	0.09
Period	8	-412	840	0.05	8	-453	921	0.04
Cohort	21	-360	761	0.17	21	-406	854	0.14
Age+period	21	-348	739	0.19	21	-403	849	0.15
Age+cohort	34	-307	683	0.29	34	-323	714	0.32
Period+cohort	27	-353	760	0.18	27	-400	854	0.15
Age+period+cohort (intrisic estimator)	38	-290	656	n/a	38	-309	694	n/a

df, degrees of freedom; AIC, Akaike information criterion
* In order to allow the age-cohort model for female more deprived to converge we replaced a zero with a 1 in the numerator of the first cohort.

Interpretation of intrinsic estimator coefficients

IE Coefficients are interpreted as follows for age, period and cohort:

1) Take the exponential of the coefficient (eg in Figure 4 and Table 1 of Additional file 3, the coefficient for females aged 50-54 is 0.737 so the age effect for females aged 50-54 is exp0.737 = 2.09)
2) The resulting number is an incident rate ratio (IRR) (eg 2.09 means that females aged 50-54 years are 109% more likely to have a drug-related death than the reference category of females.
3) The reference category is the mean effect of all ages, periods or cohorts combined (eg the reference category for the age effect for females aged 50-54 years is the mean effect of all age groups for females).
4) A negative coefficient returns an IRR below 1 (less likely than the mean to have a drug-related death) and a coefficient of 0 will return an IRR of 1 (likelihood of having a drug-related death is equal to the mean).
