# Supplementary material for: Drug-related deaths in Scotland 1979–2013: evidence of a vulnerable cohort of young men living in deprived areas
Source: BMC Public Health. 2018 Mar 27;18:357. doi: 10.1186/s12889-018-5267-2 (PMC5870372; doi:10.1186/s12889-018-5267-2)

Additional file 3: Intrinsic estimator statistics

Table 1 and Table 2 present the IE estimator coefficient statistics for females and males from the APC negative binomial models.

Table 1 Intrinsic estimator coefficient statistics for age, period and birth cohorts for drug-related deaths in Scotland for females

numerator	IE coeff	Std. Err.	z	P>z	95% CI Lower	Upper
**Age (years)**						
15-19	-0.831	0.094	-8.84	0.000	-1.015	-0.647
20-24	-0.312	0.077	-4.02	0.000	-0.464	-0.160
24-29	0.082	0.072	1.14	0.255	-0.059	0.223
30-34	0.280	0.070	3.98	0.000	0.142	0.418
35-39	0.481	0.070	6.89	0.000	0.344	0.618
40-44	0.592	0.071	8.29	0.000	0.452	0.732
45-49	0.736	0.073	10.11	0.000	0.593	0.878
50-54	0.737	0.075	9.78	0.000	0.589	0.884
55-59	0.494	0.080	6.19	0.000	0.337	0.650
60-64	0.317	0.083	3.81	0.000	0.154	0.481
65-69	0.164	0.087	1.88	0.060	-0.007	0.336
70-74	-0.275	0.097	-2.83	0.005	-0.465	-0.085
75-79	-0.485	0.106	-4.57	0.000	-0.693	-0.277
80-84	-0.861	0.129	-6.69	0.000	-1.113	-0.609
85-89	-1.119	0.177	-6.33	0.000	-1.466	-0.773
**Period**						
1979-1983	0.185	0.049	3.78	0.000	0.089	0.281
1984-1988	-0.098	0.053	-1.85	0.065	-0.201	0.006
1989-1993	-0.078	0.052	-1.51	0.130	-0.180	0.023
1994-1998	0.059	0.049	1.20	0.230	-0.037	0.156
1999-2003	-0.043	0.050	-0.86	0.389	-0.140	0.054
2004-2008	-0.086	0.051	-1.70	0.090	-0.185	0.013
2009-2013	0.060	0.053	1.14	0.256	-0.044	0.164
**Cohort**						
1894	0.570	0.404	1.41	0.159	-0.222	1.361
1899	0.352	0.249	1.41	0.158	-0.137	0.840
1904	0.130	0.185	0.70	0.483	-0.233	0.493
1909	0.259	0.143	1.81	0.070	-0.021	0.539
1914	-0.046	0.130	-0.36	0.721	-0.302	0.209
1919	-0.009	0.114	-0.08	0.939	-0.233	0.216
1924	-0.081	0.103	-0.78	0.435	-0.283	0.122
1929	-0.363	0.102	-3.58	0.000	-0.562	-0.164
1934	-0.546	0.098	-5.55	0.000	-0.739	-0.353
1939	-0.616	0.095	-6.52	0.000	-0.801	-0.431
1944	-0.611	0.089	-6.88	0.000	-0.785	-0.437
1949	-0.496	0.082	-6.03	0.000	-0.658	-0.335
1954	-0.456	0.079	-5.75	0.000	-0.612	-0.301
1959	-0.298	0.074	-4.03	0.000	-0.444	-0.153
1964	-0.079	0.070	-1.13	0.259	-0.217	0.058
1969	0.189	0.073	2.58	0.010	0.045	0.333
1974	0.492	0.079	6.25	0.000	0.338	0.646
1979	0.551	0.089	6.19	0.000	0.376	0.726
1984	0.594	0.106	5.63	0.000	0.387	0.801
1989	0.240	0.143	1.68	0.093	-0.040	0.520
1994	0.227	0.239	0.95	0.341	-0.240	0.695
	-9.644	0.034	-281.00	0.000	-9.711	-9.577
ln(denom)	1	(exposure)				

Table 2 Intrinsic estimator coefficient statistics for age, period and birth cohorts for drug-related deaths in Scotland for males

numerator	IE coeff	Std. Err.	z	P>z	95% CI Lower	Upper
**Age (years)**						
15-19	-0.796	0.095	-8.35	0.000	-0.983	-0.609
20-24	0.090	0.079	1.14	0.255	-0.065	0.246
24-29	0.465	0.072	6.44	0.000	0.324	0.607
30-34	0.580	0.069	8.43	0.000	0.445	0.714
35-39	0.677	0.068	10.03	0.000	0.545	0.810
40-44	0.808	0.068	11.95	0.000	0.675	0.940
45-49	0.687	0.071	9.67	0.000	0.548	0.826
50-54	0.501	0.076	6.58	0.000	0.352	0.650
55-59	0.241	0.083	2.92	0.004	0.079	0.403
60-64	0.092	0.089	1.03	0.303	-0.083	0.267
65-69	-0.183	0.099	-1.84	0.065	-0.377	0.012
70-74	-0.501	0.110	-4.55	0.000	-0.718	-0.285
75-79	-0.634	0.125	-5.09	0.000	-0.879	-0.390
80-84	-0.875	0.153	-5.71	0.000	-1.176	-0.575
85-89	-1.150	0.230	-5.00	0.000	-1.602	-0.699
**Period**						
1979-1983	-0.207	0.055	-3.75	0.000	-0.315	-0.099
1984-1988	-0.402	0.056	-7.16	0.000	-0.512	-0.292
1989-1993	-0.146	0.050	-2.90	0.004	-0.245	-0.047
1994-1998	0.111	0.046	2.41	0.016	0.021	0.202
1999-2003	0.157	0.046	3.43	0.001	0.067	0.246
2004-2008	0.221	0.048	4.57	0.000	0.126	0.315
2009-2013	0.266	0.055	4.87	0.000	0.159	0.373
**Cohort**						
1894	0.642	0.651	0.99	0.324	-0.634	1.918
1899	0.347	0.373	0.93	0.351	-0.383	1.078
1904	0.170	0.249	0.68	0.495	-0.318	0.659
1909	0.458	0.176	2.60	0.009	0.112	0.803
1914	-0.185	0.169	-1.09	0.274	-0.516	0.146
1919	-0.482	0.154	-3.14	0.002	-0.784	-0.181
1924	-0.324	0.127	-2.54	0.011	-0.573	-0.074
1929	-0.551	0.120	-4.61	0.000	-0.785	-0.317
1934	-0.743	0.112	-6.61	0.000	-0.963	-0.522
1939	-0.722	0.102	-7.07	0.000	-0.922	-0.522
1944	-0.864	0.095	-9.13	0.000	-1.049	-0.679
1949	-0.683	0.084	-8.18	0.000	-0.847	-0.519
1954	-0.542	0.076	-7.12	0.000	-0.691	-0.393
1959	-0.299	0.068	-4.38	0.000	-0.433	-0.165
1964	0.100	0.062	1.60	0.109	-0.022	0.222
1969	0.470	0.064	7.36	0.000	0.345	0.596
1974	0.844	0.068	12.35	0.000	0.710	0.978
1979	0.948	0.078	12.21	0.000	0.795	1.100
1984	0.807	0.093	8.69	0.000	0.625	0.989
1989	0.429	0.121	3.55	0.000	0.192	0.666
1994	0.180	0.205	0.88	0.380	-0.222	0.581
	-9.232	0.046	-198.96	0.000	-9.323	-9.141
ln(denom)	1	(exposure)				

Figure 1 shows the IE coefficients together with upper and lower 95% confidence intervals for females and males.

Figure 1 Intrinsic estimator coefficients with 95% confidence intervals for age, period and birth cohorts for drug-related deaths in Scotland stratified by sex (females on left and males on the right).

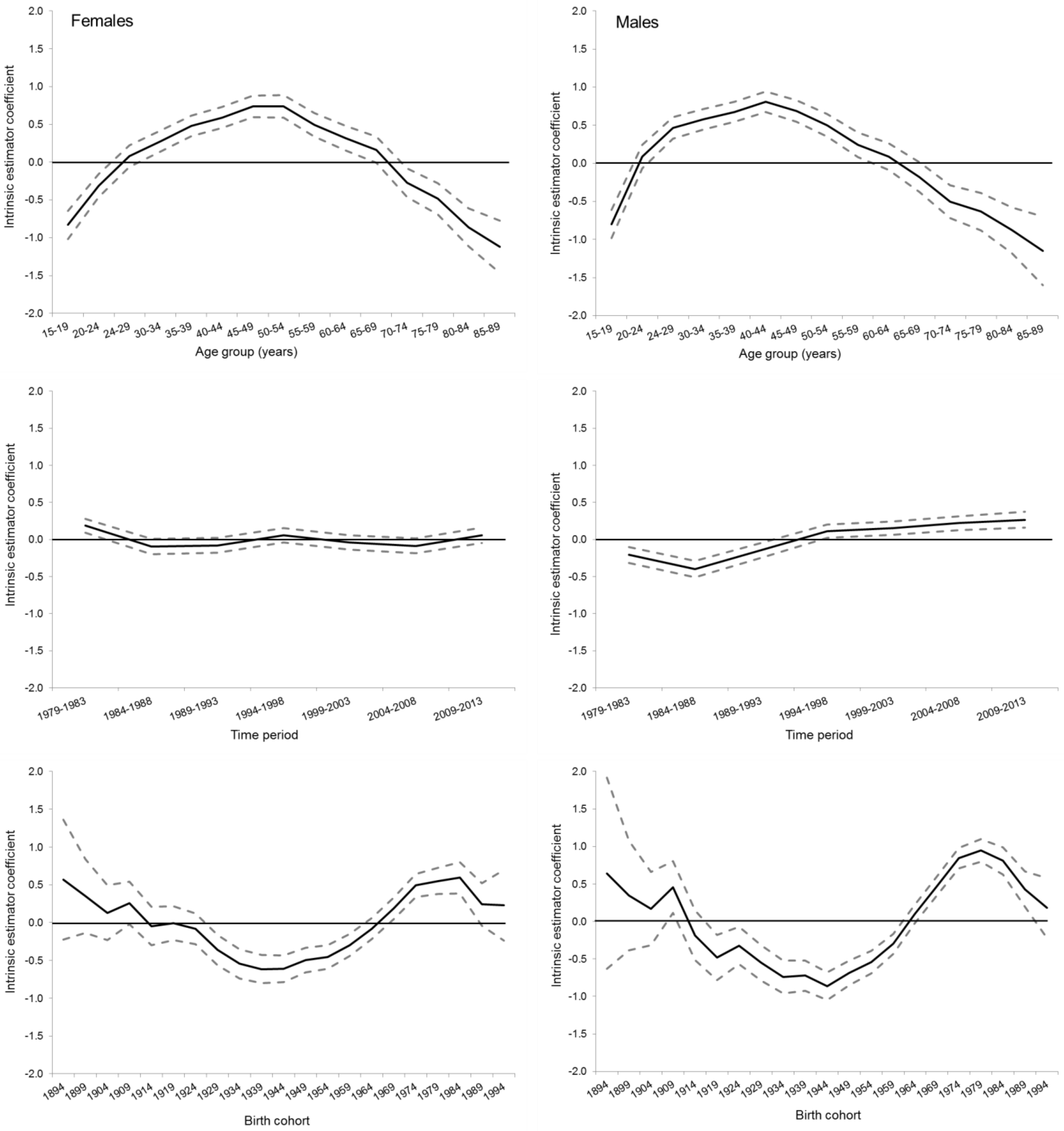

Tables 3-6 present the IE estimator coefficient statistics for females and males from the APC negative binomial models stratified by Carstairs deprivation.

*Table 3 Intrinsic estimator coefficient statistics for age, period and birth cohorts for drug-related deaths in Scotland for females living in the most deprived quintile**

Numerator	IE coeff	Std. Err.	z	P>z	95% Lower	Upper
**Age (years)**						
15-19	-0.684	0.166	-4.120	0.000	-1.009	-0.359
20-24	-0.102	0.132	-0.780	0.437	-0.361	0.156
24-29	0.236	0.120	1.970	0.049	0.001	0.472
30-34	0.363	0.115	3.160	0.002	0.138	0.589
35-39	0.631	0.112	5.660	0.000	0.413	0.850
40-44	0.754	0.112	6.730	0.000	0.535	0.974
45-49	0.722	0.118	6.120	0.000	0.491	0.953
50-54	0.679	0.122	5.550	0.000	0.439	0.918
55-59	0.447	0.132	3.390	0.001	0.189	0.705
60-64	0.095	0.146	0.650	0.515	-0.190	0.380
65-69	-0.071	0.155	-0.460	0.647	-0.376	0.233
70-74	-0.911	0.196	-4.660	0.000	-1.295	-0.528
75-79	-1.209	0.227	-5.320	0.000	-1.654	-0.763
80-84	-0.950	0.239	-3.970	0.000	-1.420	-0.481
**Period**						
1979-1983	-0.167	0.094	-1.770	0.077	-0.351	0.018
1984-1988	-0.467	0.100	-4.690	0.000	-0.662	-0.271
1989-1993	-0.184	0.088	-2.090	0.037	-0.357	-0.011
1994-1998	0.179	0.078	2.290	0.022	0.026	0.333
1999-2003	0.144	0.079	1.830	0.067	-0.010	0.298
2004-2008	0.177	0.081	2.180	0.029	0.018	0.337
2009-2013	0.317	0.090	3.510	0.000	0.140	0.493
**Cohort**						
1899	-0.481	0.889	-0.540	0.588	-2.223	1.261
1904	-0.417	0.653	-0.640	0.523	-1.697	0.862
1909	0.997	0.299	3.330	0.001	0.411	1.583
1914	0.148	0.282	0.520	0.601	-0.406	0.701
1919	-0.055	0.251	-0.220	0.827	-0.546	0.437
1924	0.164	0.206	0.800	0.426	-0.239	0.566
1929	-0.163	0.193	-0.840	0.399	-0.541	0.216
1934	-0.256	0.181	-1.410	0.157	-0.611	0.099
1939	-0.587	0.177	-3.310	0.001	-0.935	-0.240
1944	-0.490	0.160	-3.060	0.002	-0.804	-0.177
1949	-0.314	0.144	-2.190	0.029	-0.595	-0.032
1954	-0.404	0.135	-3.000	0.003	-0.668	-0.140
1959	-0.161	0.119	-1.350	0.178	-0.394	0.073
1964	0.080	0.109	0.740	0.462	-0.134	0.294
1969	0.399	0.109	3.660	0.000	0.186	0.613
1974	0.562	0.116	4.850	0.000	0.335	0.789
1979	0.437	0.133	3.280	0.001	0.176	0.699
1984	0.349	0.163	2.140	0.032	0.030	0.668
1989	0.144	0.212	0.680	0.497	-0.272	0.560
1994	0.048	0.370	0.130	0.897	-0.678	0.774
_cons	-9.302	0.075	-124.620	0.000	-9.448	-9.155
ln(denom)	1	(exposure)				

* The data for the oldest birth cohort in females in the most deprived group was substituted with that for the 1904 cohort

Table 4 Intrinsic estimator coefficient statistics for age, period and birth cohorts for drug-related deaths in Scotland for females living in the less deprived four quintiles

Numerator	IE coeff	Std. Err.	z	P>z	95% Lower	95% Upper
**Age (years)**						
15-19	-0.800	0.096	-8.300	0.000	-0.988	-0.611
20-24	-0.395	0.079	-4.980	0.000	-0.551	-0.240
24-29	-0.042	0.072	-0.580	0.563	-0.183	0.100
30-34	0.193	0.069	2.810	0.005	0.058	0.328
35-39	0.384	0.069	5.580	0.000	0.249	0.520
40-44	0.426	0.071	6.010	0.000	0.287	0.564
45-49	0.665	0.071	9.420	0.000	0.527	0.803
50-54	0.623	0.073	8.540	0.000	0.480	0.766
55-59	0.402	0.079	5.110	0.000	0.248	0.557
60-64	0.223	0.082	2.730	0.006	0.063	0.383
65-69	0.061	0.086	0.710	0.476	-0.107	0.230
70-74	-0.244	0.095	-2.560	0.010	-0.430	-0.057
75-79	-0.475	0.105	-4.520	0.000	-0.681	-0.269
80-84	-1.022	0.150	-6.830	0.000	-1.315	-0.729
**Period**						
1979-1983	0.245	0.048	5.130	0.000	0.152	0.339
1984-1988	-0.031	0.053	-0.580	0.565	-0.135	0.074
1989-1993	-0.110	0.054	-2.010	0.044	-0.216	-0.003
1994-1998	-0.010	0.052	-0.190	0.846	-0.111	0.091
1999-2003	-0.050	0.051	-0.990	0.324	-0.150	0.050
2004-2008	-0.136	0.052	-2.620	0.009	-0.237	-0.034
2009-2013	0.090	0.051	1.760	0.078	-0.010	0.191
**Cohort**						
1899	0.660	0.296	2.230	0.026	0.080	1.240
1904	0.392	0.188	2.080	0.037	0.023	0.761
1909	0.270	0.147	1.840	0.066	-0.018	0.557
1914	0.136	0.125	1.090	0.275	-0.108	0.381
1919	0.179	0.107	1.660	0.096	-0.032	0.389
1924	0.045	0.099	0.450	0.653	-0.150	0.240
1929	-0.215	0.095	-2.250	0.024	-0.401	-0.028
1934	-0.506	0.097	-5.240	0.000	-0.695	-0.317
1939	-0.498	0.092	-5.400	0.000	-0.678	-0.317
1944	-0.537	0.088	-6.100	0.000	-0.710	-0.365
1949	-0.421	0.081	-5.180	0.000	-0.580	-0.262
1954	-0.436	0.080	-5.480	0.000	-0.593	-0.280
1959	-0.297	0.075	-3.950	0.000	-0.444	-0.149
1964	-0.132	0.072	-1.830	0.067	-0.274	0.009
1969	0.024	0.077	0.310	0.759	-0.128	0.175
1974	0.266	0.082	3.240	0.001	0.105	0.427
1979	0.456	0.090	5.050	0.000	0.279	0.633
1984	0.562	0.105	5.330	0.000	0.355	0.769
1989	0.019	0.159	0.120	0.903	-0.292	0.331
1994	0.033	0.263	0.120	0.901	-0.484	0.549
_cons	-9.790	0.031	-313.770	0.000	-9.851	-9.729
ln(denom)	1	(exposure)				

Table 5 Intrinsic estimator coefficient statistics for age, period and birth cohorts for drug-related deaths in Scotland for males living in the most deprived quintile

Numerator	IE coeff	Std. Err.	z	P>z	95% Lower	95% Upper
**Age (years)**						
15-19	-0.766	0.140	-5.460	0.000	-1.041	-0.491
20-24	0.060	0.116	0.520	0.603	-0.166	0.287
24-29	0.419	0.109	3.860	0.000	0.206	0.632
30-34	0.640	0.107	6.000	0.000	0.431	0.849
35-39	0.717	0.109	6.550	0.000	0.503	0.932
40-44	0.864	0.112	7.730	0.000	0.645	1.083
45-49	0.600	0.123	4.880	0.000	0.359	0.840
50-54	0.390	0.133	2.930	0.003	0.129	0.650
55-59	0.403	0.140	2.890	0.004	0.130	0.677
60-64	0.036	0.155	0.230	0.817	-0.268	0.340
65-69	-0.591	0.191	-3.090	0.002	-0.965	-0.216
70-74	-0.425	0.188	-2.260	0.024	-0.794	-0.057
75-79	-1.195	0.256	-4.670	0.000	-1.696	-0.693
80-84	-1.152	0.311	-3.710	0.000	-1.761	-0.543
**Period**						
1979-1983	-0.351	0.093	-3.760	0.000	-0.534	-0.168
1984-1988	-0.598	0.101	-5.940	0.000	-0.796	-0.401
1989-1993	-0.194	0.088	-2.190	0.028	-0.367	-0.020
1994-1998	0.209	0.079	2.640	0.008	0.054	0.364
1999-2003	0.304	0.077	3.960	0.000	0.154	0.455
2004-2008	0.263	0.081	3.250	0.001	0.104	0.421
2009-2013	0.367	0.089	4.120	0.000	0.193	0.541
**Cohort**						
1899	1.048	0.694	1.510	0.131	-0.311	2.408
1904	0.823	0.459	1.790	0.073	-0.077	1.722
1909	0.308	0.340	0.910	0.364	-0.357	0.974
1914	-0.373	0.325	-1.150	0.251	-1.010	0.264
1919	-0.487	0.264	-1.850	0.065	-1.003	0.030
1924	-0.438	0.213	-2.050	0.040	-0.856	-0.020
1929	-0.826	0.205	-4.030	0.000	-1.228	-0.424
1934	-0.948	0.192	-4.930	0.000	-1.325	-0.571
1939	-0.768	0.168	-4.560	0.000	-1.098	-0.437
1944	-0.820	0.159	-5.140	0.000	-1.132	-0.507
1949	-0.593	0.140	-4.230	0.000	-0.868	-0.318
1954	-0.497	0.129	-3.850	0.000	-0.750	-0.244
1959	-0.130	0.116	-1.130	0.260	-0.357	0.096
1964	0.344	0.105	3.270	0.001	0.138	0.550
1969	0.706	0.107	6.620	0.000	0.497	0.915
1974	0.865	0.114	7.620	0.000	0.642	1.087
1979	0.839	0.127	6.600	0.000	0.590	1.088
1984	0.725	0.150	4.820	0.000	0.430	1.020
1989	0.295	0.196	1.510	0.132	-0.088	0.679
1994	-0.074	0.343	-0.220	0.829	-0.746	0.598
_cons	-8.684	0.062	-139.250	0.000	-8.806	-8.562
ln(denom)	1	(exposure)				

Table 6 Intrinsic estimator coefficient statistics for age, period and birth cohorts for drug-related deaths in Scotland for males living in the less deprived four quintiles

Numerator	IE coeff	Std. Err.	z	P>z	95% Lower	95% Upper
**Age (years)**						
15-19	-0.776	0.086	-8.970	0.000	-0.945	-0.606
20-24	0.122	0.067	1.810	0.070	-0.010	0.254
24-29	0.419	0.062	6.760	0.000	0.297	0.540
30-34	0.418	0.061	6.910	0.000	0.299	0.537
35-39	0.588	0.061	9.640	0.000	0.469	0.708
40-44	0.646	0.063	10.250	0.000	0.522	0.769
45-49	0.640	0.067	9.510	0.000	0.508	0.772
50-54	0.419	0.074	5.680	0.000	0.274	0.563
55-59	0.055	0.084	0.650	0.513	-0.110	0.220
60-64	-0.040	0.089	-0.450	0.651	-0.216	0.135
65-69	-0.187	0.096	-1.940	0.052	-0.376	0.002
70-74	-0.662	0.114	-5.800	0.000	-0.885	-0.438
75-79	-0.688	0.123	-5.600	0.000	-0.929	-0.447
80-84	-0.953	0.164	-5.810	0.000	-1.275	-0.632
**Period**						
1979-1983	-0.220	0.054	-4.070	0.000	-0.327	-0.114
1984-1988	-0.374	0.057	-6.580	0.000	-0.486	-0.263
1989-1993	-0.183	0.051	-3.560	0.000	-0.284	-0.082
1994-1998	0.073	0.046	1.600	0.110	-0.016	0.162
1999-2003	0.136	0.044	3.130	0.002	0.051	0.222
2004-2008	0.271	0.044	6.160	0.000	0.184	0.357
2009-2013	0.298	0.049	6.050	0.000	0.201	0.395
**Cohort**						
1899	0.443	0.458	0.970	0.333	-0.455	1.341
1904	0.285	0.273	1.040	0.297	-0.251	0.820
1909	0.664	0.176	3.760	0.000	0.318	1.009
1914	0.028	0.165	0.170	0.866	-0.295	0.350
1919	-0.285	0.150	-1.900	0.058	-0.580	0.009
1924	-0.155	0.123	-1.260	0.208	-0.396	0.086
1929	-0.353	0.111	-3.180	0.001	-0.571	-0.136
1934	-0.583	0.107	-5.460	0.000	-0.792	-0.374
1939	-0.643	0.099	-6.500	0.000	-0.838	-0.449
1944	-0.820	0.093	-8.790	0.000	-1.003	-0.637
1949	-0.622	0.082	-7.630	0.000	-0.782	-0.463
1954	-0.572	0.075	-7.640	0.000	-0.719	-0.425
1959	-0.384	0.067	-5.760	0.000	-0.515	-0.254
1964	-0.036	0.060	-0.590	0.553	-0.154	0.083
1969	0.337	0.059	5.680	0.000	0.220	0.453
1974	0.713	0.061	11.720	0.000	0.593	0.832
1979	0.858	0.068	12.710	0.000	0.726	0.990
1984	0.706	0.082	8.640	0.000	0.546	0.866
1989	0.310	0.111	2.790	0.005	0.092	0.528
1994	0.112	0.204	0.550	0.582	-0.287	0.512
_cons	-9.397	0.038	-248.750	0.000	-9.471	-9.323
ln(denom)	1	(exposure)				

Figures 2 and 3 show the IE coefficients together with upper and lower 95% confidence intervals for females and males stratified by Carstairs deprivation.

*Figure 2 Intrinsic estimator coefficients with 95% confidence intervals for age, period and birth cohorts for drug-related deaths in Scotland for females stratified by deprivation (most deprived on left and less deprived on right).**

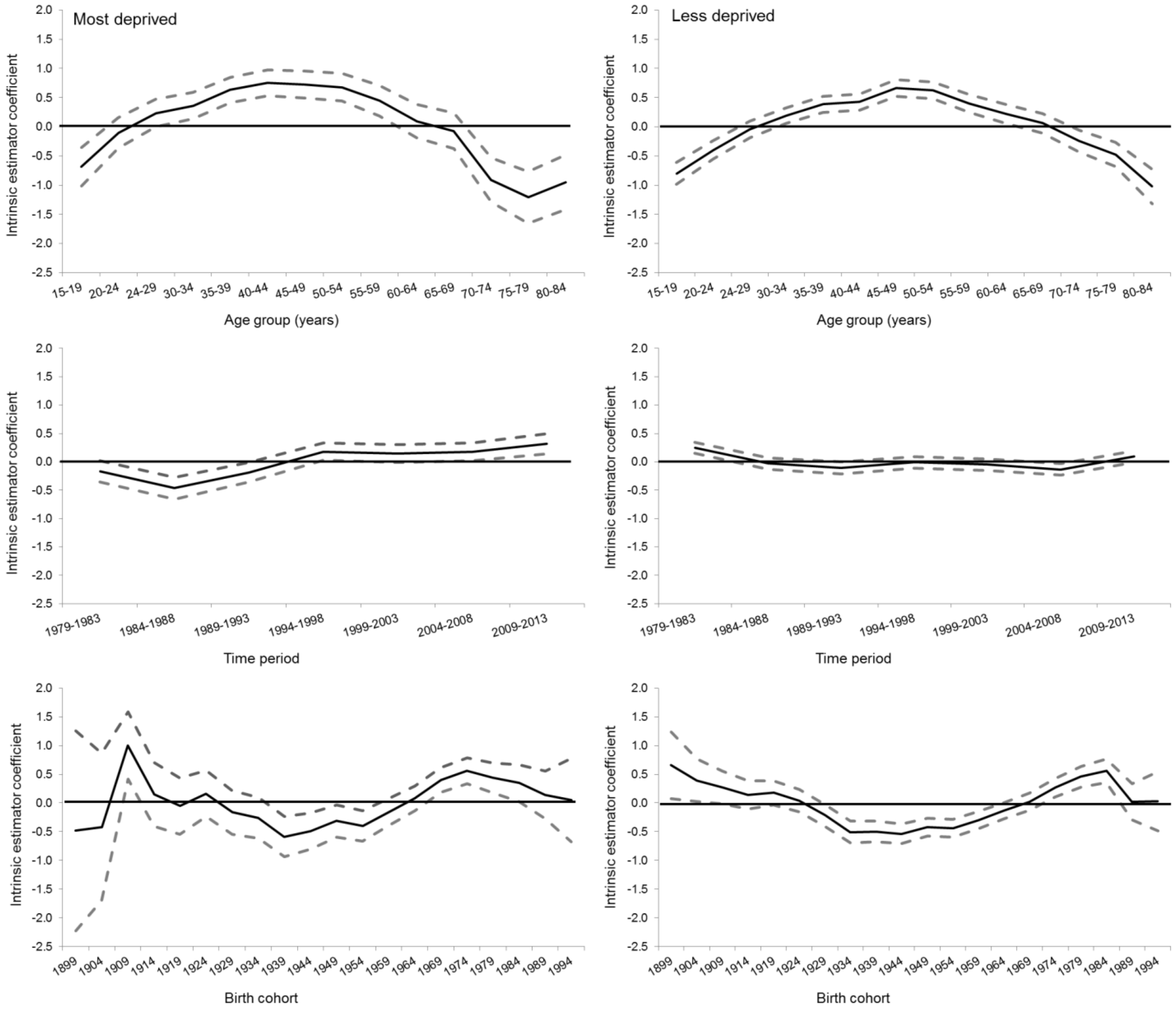

* The data for the oldest birth cohort in females in the most deprived group was substituted with that for the 1904 cohort

Figure 3 Intrinsic estimator coefficients with 95% confidence intervals for age, period and birth cohorts for drug-related deaths in Scotland for males stratified by deprivation (most deprived on left and less deprived on right).

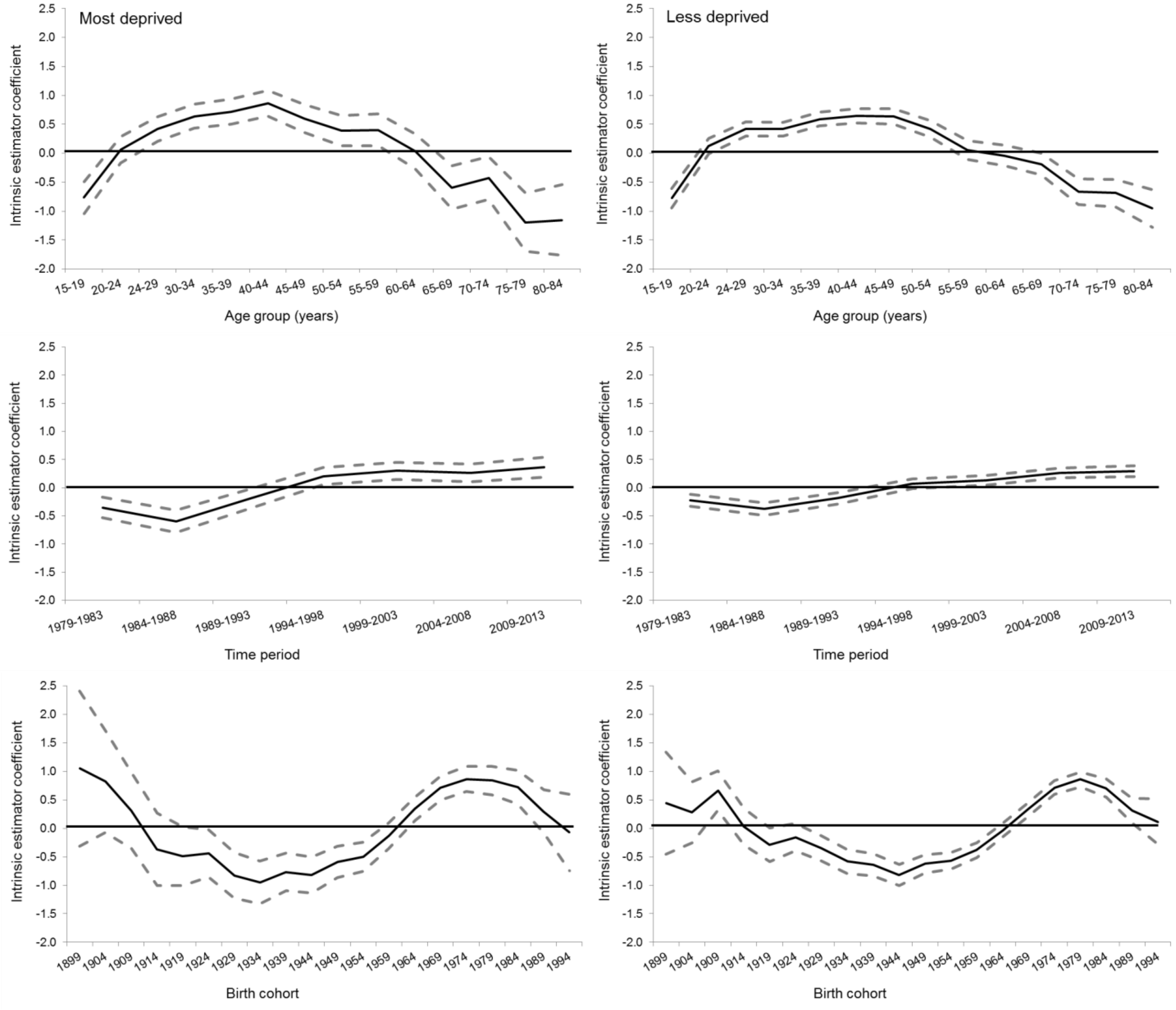

Supplement: Supplementary file 3 — Intrinsic estimator statistics. Table S1. Intrinsic estimator coefficient statistics for age, period and birth cohorts for drug-related deaths in Scotland for females. Table S2. Intrinsic estimator coefficient statistics for age, period and birth cohorts for drug-related deaths in Scotland for males. Figure S1. Intrinsic estimator coefficients with 95% confidence intervals for age, period and birth cohorts for drug-related deaths in Scotland stratified by sex. Table S3. Intrinsic estimator coefficient statistics for age, period and birth cohorts for drug-related deaths in Scotland for females living in the most deprived quintile. Table S4. Intrinsic estimator coefficient statistics for age, period and birth cohorts for drug-related deaths in Scotland for females living in the less deprived four quintiles. Table S5. Intrinsic estimator coefficient statistics for age, period and birth cohorts for drug-related deaths in Scotland for males living in the most deprived quintile. Table S6. Intrinsic estimator coefficient statistics for age, period and birth cohorts for drug-related deaths in Scotland for males living in the less deprived four quintiles. Figure S2. Intrinsic estimator coefficients with 95% confidence intervals for age, period and birth cohorts for drug-related deaths in Scotland for females stratified by deprivation. Figure S3. Intrinsic estimator coefficients with 95% confidence intervals for age, period and birth cohorts for drug-related deaths in Scotland for males stratified by deprivation. (PDF 1151 kb) [file 12889_2018_5267_MOESM3_ESM.pdf]
